# TGFβ1 orchestrates renal fibrosis following *Escherichia coli* pyelonephritis

**DOI:** 10.14814/phy2.14401

**Published:** 2020-03-30

**Authors:** Teri N. Hreha, Christina A. Collins, Allyssa L. Daugherty, Joy Twentyman, Nitin Paluri, David A. Hunstad

**Affiliations:** ^1^ Department of Pediatrics Washington University School of Medicine St. Louis MO USA; ^2^ Department of Molecular Microbiology Washington University School of Medicine St. Louis MO USA; ^3^ Present address: Department of Global Health University of Washington Seattle WA USA

**Keywords:** *Escherichia coli*, fibrosis, pyelonephritis, renal scarring, TGFβ

## Abstract

Renal scarring after pyelonephritis is linked to long‐term health risks for hypertension and chronic kidney disease. Androgen exposure increases susceptibility to, and severity of, uropathogenic *Escherichia coli* (UPEC) pyelonephritis and resultant scarring in both male and female mice, while anti‐androgen therapy is protective against severe urinary tract infection (UTI) in these models. This work employed androgenized female C57BL/6 mice to elucidate the molecular mechanisms of post‐infectious renal fibrosis and to determine how these pathways are altered by the presence of androgens. We found that elevated circulating testosterone levels primed the kidney for fibrosis by increasing local production of TGFβ1 before the initiation of UTI, altering the ratio of transcription factors Smad2 and Smad3 and increasing the presence of mesenchymal stem cell (MSC)‐like cells and Gli1 + activated myofibroblasts, the cells primarily responsible for deposition of scar components. Increased production of TGFβ1 and aberrations in Smad2:Smad3 were maintained throughout the course of infection in the presence of androgen, correlating with renal scarring that was not observed in non‐androgenized female mice. Pharmacologic inhibition of TGFβ1 signaling blunted myofibroblast activation. We conclude that renal fibrosis after pyelonephritis is exacerbated by the presence of androgens and involves activation of the TGFβ1 signaling cascade, leading to increases in cortical populations of MSC‐like cells and the Gli1 + activated myofibroblasts that are responsible for scarring.

## Graphical abstract

Circulating androgens prime the kidney for fibrosis upon infectious injury (*Escherichia coli* pyelonephritis) by enhancing local TGFβ1 production and myofibroblast activation.

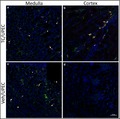

## 1 INTRODUCTION

Urinary tract infection (UTI) affects millions of people worldwide, exerting substantial impact both economically and on quality of life. Uropathogenic *Escherichia coli* (UPEC) cause over 80% of UTIs, including acute and recurrent bladder infection (cystitis) and ascending infection of one or both kidneys (pyelonephritis). Epidemiologically, UTI is primarily a disease of females; however, infant boys and elderly men exhibit higher rates of UTI compared to females of equivalent ages (Foxman, 2002; Foxman, Barlow, D’Arcy, Gillespie, & Sobel, 2000; Hummers‐Pradier, Ohse, Koch, Heizmann, & Kochen, 2004; Ruben et al., 1995; Shaikh, Morone, Bost, & Farrell, 2008; Wettergren, Jodal, & Jonasson, 1985). Furthermore, complicated UTI in males carries increased morbidity and mortality compared to that of females (Foxman, 2002; Lipsky, 1989). In childhood, ~7% of febrile UTI episodes result in formation of renal scars (Shaikh et al., 2016), which correlate with elevated risk for hypertension, chronic kidney disease, and end‐stage kidney disease later in life (Calderon‐Margalit et al., 2018; Efstathiou et al., 2003; Foxman, Klemstine, & Brown, 2003; Ki, Park, Choi, & Foxman, 2004; Nicolle, Friesen, Harding, & Roos, 1996; Ricardo et al., 2019).

Testosterone signaling through the androgen receptor increases susceptibility to, and severity of, experimental UPEC pyelonephritis in both male and female mice (Olson, Hruska, & Hunstad, 2016), while anti‐androgen therapy is protective against severe UTI in mice (Olson et al., 2017) and in women with polycystic ovary syndrome (Gamal, Elkholi, & Nagy, 2016; Wang, Su, Liu, Chang, & Chen, 2005), a common hyperandrogenic condition. As observed in children, mice with severe pyelonephritis develop renal fibrosis and scarring (Olson et al., 2018). In non‐infectious models of renal injury, fibrosis is caused by excess extracellular matrix (ECM) deposition by α‐smooth muscle actin (αSMA)‐positive activated myofibroblasts. Just over half of activated myofibroblasts are derived from Gli1+ mesenchymal stem cell (MSC)‐like cells that reside in the perivascular niche (Kramann, Fleig, et al., 2015; Kramann, Schneider, et al., 2015). The remaining activated myofibroblasts in the recently injured kidney may be derived from several different progenitor cell types including resident fibroblasts, epithelial cells, endothelial cells, or precursors recruited from the bone marrow (Chen et al., 2011; Iwano et al., 2002; Ng et al., 1998; Piera‐Velazquez, Li, & Jimenez, 2011; Reich et al., 2013; Schnaper, Hayashida, Hubchak, & Poncelet, 2003; Wu et al., 2013; Xavier et al., 2015). However, activated myofibroblasts can lose their progenitor cell type‐specific markers during transition, making it challenging to definitively determine their cell lineage (Humphreys, 2018).

Activated myofibroblasts are highly sensitive to TGFβ1 signaling, the production of which is known to be upregulated following experimental non‐infectious renal injury (Gentle et al., 2013; Loeffler & Wolf, 2014). Ligation of the TGFβ1 receptor leads to phosphorylation of the transcription factors Smad2 and Smad3, which then complex with Smad4 and translocate into the nucleus, upregulating transcription of αSMA and ECM components such as collagens I and III (Hill, 2009; Inman, Nicolás, & Hill, 2002). Other TGFβ superfamily members (e.g., activin A), as well as members of the Hedgehog signaling cascade (e.g., Gli1), can also signal through Smad transcription factors using nonredundant receptors and pathways (Agapova, Fang, Sugatani, Seifert, & Hruska, 2016; Kramann, 2016; Yamashita, Maeshima, Kojima, & Nojima, 2004; Zhang, Tian, & Xing, 2016). Under normal conditions, a renal insult subsides and the secreted ECM is subsequently degraded by locally generated collagenases (Bauman et al., 2010; Wang et al., 2006; Zeisberg et al., 2003), enabling productive healing and regeneration of functional tubules in the affected area of kidney. Aberrations in this signaling cascade, or more severe or prolonged injury, may push fibrotic signaling beyond the point of no return, precluding productive repair and resulting in permanent scarring (Genovese, Manresa, Leeming, Karsdal, & Boor, 2014).

We recently developed a preclinical model in which experimental pyelonephritis (even when successfully treated with clinically relevant antibiotics) results in scar formation in C3H mice, a strain featuring vesicoureteral reflux (Olson et al., 2017). Here, we extended these models into non‐refluxing C57BL/6 mice, which when androgenized are permissive for pyelonephritis, but without the abscess formation seen in C3H mice (Olson et al., 2018). This system allowed us to interrogate the molecular mechanisms of renal fibrosis, separate from the severity of UTI per se, and to specify how the activity of these pathways is enhanced in the presence of androgens.

## 2 METHODS

### 2.1 Bacterial strains

Uropathogenic *Escherichia coli* (UPEC) strain UTI89, a clinical cystitis isolate (Chen et al., 2009), was grown in Luria‐Bertani (LB; Becton Dickinson [BD]) broth statically overnight at 37°C. Cultures were centrifuged for 10 min at 7,500*g* at 4°C before being resuspended in sterile phosphate‐buffered saline (PBS) to a density of ~4 × 10^8^ colony‐forming units (CFU)/ml.

### 2.2 Animals

All animal protocols received prior approval from the Washington University Institutional Animal Care and Use Committee. Experiments were conducted in C57BL/6 mice (#000664; Jackson Laboratories) or, for immunofluorescence studies, in bigenic Gli1‐tdTomato^+^ mice harboring a tamoxifen‐inducible Cre that enables tdTomato production from the Gli1 promoter (kind gift of B. Humphreys; (Kramann, Schneider, et al., 2015)). For androgenization, female mice of either strain were administered testosterone cypionate (TC; Depo‐Testosterone, Pfizer) 150 mg/kg IM weekly beginning at 5 weeks of age and continuing until sacrifice. UTI was initiated by bladder inoculation with 1–2 × 10^7^ CFU of UPEC via catheter at 7 weeks of age, as described previously (Hannan & Hunstad, 2016; Hung, Dodson, & Hultgren, 2009). Experiments were conducted similarly in Gli1‐tdTomato^+^ mice, with the addition of three doses of tamoxifen (0.1 mg/kg IP in corn oil/3% ethanol; Sigma) ending 10 days before UTI initiation.

### 2.3 TGFβ1 receptor inhibition experiments

For TGFβ1 receptor inhibition experiments, mice were treated as described above, and treated with the ALK5‐specific inhibitor GW788388 (Gellibert et al., 2006) (Selleck Chemicals), 3 mg/kg IP with 6% DMSO in PBS, daily for 7 days beginning 1 day prior to UTI initiation. Mice were sacrificed 7 dpi as described below.

### 2.4 Determination of bacterial loads

At the indicated time points, mice were terminally anesthetized with inhaled isoflurane (Patterson Veterinary, Greeley, CO) and perfused with 4°C PBS via the left ventricle. Bladders and kidneys were aseptically removed and homogenized in 4°C PBS; tissue homogenates were serially diluted and plated on LB agar.

### 2.5 Serum analysis

Blood was collected by submandibular puncture, or by cardiac puncture for collections at the time of sacrifice, into BD Microtainer serum separator tubes and centrifuged at 10,000*g*. Serum testosterone and estradiol measurements were performed by enzyme immunoassay at the Ligand Assay and Analysis Core, University of Virginia Center for Research in Reproduction.

### 2.6 Quantitative RT‐PCR

At various time points, mRNA was extracted from flash‐frozen kidneys with RNA Stat‐60 (Amsbio), according to manufacturer's instructions. 1 µg mRNA was converted to cDNA with the iScript cDNA Synthesis Kit (Bio‐Rad) according to the manufacturer's instructions. Roughly 20 ng of cDNA was run in triplicate with SsoAdvanced Universal SYBR Green Supermix (Bio‐Rad) containing 350 nM primers. Thermal cycling was performed using an Applied Biosystems Fast 7500 RT‐PCR system with the following protocol: 95°C, 3 min; 40 × (95°C, 10 s; 60°C, 30 s). Primers used in this study are provided in Table S1.

### 2.7 Tissue preparation and histology

Mice were euthanized as described above, and excised kidneys were fixed in 4% paraformaldehyde in PBS at 4°C for 1 hr, then incubated overnight in 30% sucrose in PBS at 4°C before embedding into OCT (Fisher Scientific). Embedded tissues were cryosectioned into 5‐ to 8‐µm sections and mounted on Superfrost Plus slides (Fisher Scientific). Gomori trichrome staining was performed with the Gomori Trichrome Stain Kit (Richard‐Allan Scientific) according to the manufacturer's instructions.

For immunohistochemistry, sections were washed with Tris‐buffered saline (TBS; 50 mM Tris, 150 mM NaCl, pH 7.6), incubated with 3% H_2_O_2_ in methanol, washed with TBS, and blocked with 10% fetal bovine serum (FBS; Gibco, Dublin, Ireland) in TBS. Sections were then stained for collagen I (1:250, SouthernBiotech #1310‐01, RRID:AB_2753206), washed with TBS, and probed with 1:250 biotinylated anti‐goat IgG (Invitrogen #31730, RRID:AB_228368), then washed with TBS and probed again with 1:250 streptavidin‐HRP (ThermoFisher Scientific #N504). After washing, sections were developed with DAB (Vector Laboratories #SK‐4100), according to the manufacturer's instructions. Sections were washed again, and dyed with Gill No. 2 Hematoxylin (Sigma) before dehydration and mounting. For analysis, 10 random images of each section were taken at 10× magnification, and quantified using the IHC profiler plugin for ImageJ (National Institutes of Health; Bethesda, MD (Varghese, Bukhari, Malhotra, & De, 2014)). Representative IHC images are presented in Figure S6.

For immunofluorescence microscopy, sections were washed with PBS, blocked with 10% FBS in PBS, and stained with primary antibodies against CD140b (APB5)‐APC (1:200, eBioscience #14‐1402, RRID:AB_467493), αSMA (1A4/asm‐1)‐PerCP (1:150, Novus Biologicals #NBP2‐34522PCP), aquaporin 2 (E2)‐AlexaFluor 647 (1:200, Santa Cruz Biotechnology #sc‐515770, RRID:AB_2810957), aquaporin 1 (B‐11)‐AlexaFluor 647 (1:200, Santa Cruz Biotechnology #sc‐25287, RRID:AB_626694), Tamm‐Horsfall protein (774056) (THP, uromodulin; 1:200, ThermoFisher #MA5‐24374, RRID:AB_2606307), calbindin D28k (D‐4)‐AlexaFluor 647 (1:200, Santa Cruz Biotechnology #sc‐365360, RRID:AB_10841576), or TGFβ1 (3C11)‐AlexaFluor 594 (1:200, Santa Cruz Biotechnology #sc‐130348, RRID:AB_1567351). Sections were washed with PBS and probed when necessary with a goat anti‐rabbit AlexaFluor 488‐conjugated secondary antibody (1:300, Jackson ImmunoResearch #111‐095‐144, RRID:AB_2337978). Sections were then stained with 1:5,000 4′,6‐diamidino‐2‐phenylindole (DAPI) and mounted with ProLong Gold (both from Life Technologies). Images were captured digitally using a Zeiss LSM 880 Airyscan confocal microscope.

### 2.8 Flow cytometry

Kidneys were harvested as described above and manually homogenized into 4°C RPMI (Gibco), then treated with RBC lysis buffer (155 mM NH_4_Cl, 10 mM KHCO_3_) at room temperature to ensure lysis of any RBCs remaining after perfusion. After washing, cells were resuspended in 4°C PBS and stained with Live/Dead Fixable Yellow (ThermoFisher Scientific). After staining and washing, cells were resuspended in 4°C FACS buffer (10% FBS, 1% w/v sodium azide in PBS) and treated with Fc Block (BD Biosciences) on ice, then stained with labeled antibodies against the following extracellular antigens: E‐cadherin (CD324)‐PE‐Cy7 (1:200, Biolegend #147309, RRID:AB_2564187), nestin (Rat‐401)‐FITC (1:20, Santa Cruz Biotechnology #sc‐33677, RRID:AB_627995), CD140b (APB5)‐APC (1:20, Biolegend #136008, RRID:AB_2268091), and CD45 (30‐F11)‐BV510 (1:200, BD Biosciences #563891, RRID:AB_2734134). Cells were washed again, fixed in 4% paraformaldehyde in PBS, and permeabilized on ice with Perm/Wash buffer (10% FBS, 1% w/v sodium azide, 1.3 mM saponin in PBS, pH 7.4–7.6). The cells were then stained with the following labeled intracellular antibodies: Gli1 (C‐1)‐PE (1:25, Santa Cruz Biotechnology #sc‐515751), αSMA (1A4/asm‐1)‐PerCP (1:10, Novus Biologicals #NBP2‐34522PCP), and TGFβ1 (9016)‐AlexaFluor 700 (1:50, R&D Systems #IC240N, RRID:AB_884506). After staining, cells were washed, resuspended in FACS buffer, and subjected to flow cytometry on an LSR II Fortessa instrument (BD Biosciences); results were analyzed using FlowJo software (Tree Star Inc.). A representation of the gating scheme used for analysis is provided in Figure S7.

### 2.9 Immunoblotting

Kidneys were harvested as described above, flash frozen in liquid nitrogen, and stored at −80°C until use. They were homogenized in RIPA buffer (50 mM Tris‐HCl, 150 mM NaCl, 1% Nonidet P‐40, 0.1% SDS, 0.5% sodium deoxycholate, pH 7.4) containing PhosSTOP phosphatase inhibitor and cOmplete Mini protease inhibitor (both from Roche). Lysates were cleared by centrifugation (2 × 5 min at max speed), and total protein concentration was determined by bicinchoninic acid assay (Invitrogen). 80 µg protein per lane was loaded into SDS‐PAGE gels and transferred to PVDF membranes. Membranes were blocked with 5% milk (Carnation) in PBS with 0.05% Tween‐20 (PBST) and probed with primary antibodies for Smad2/3 (C‐8) (1:500, Santa Cruz Biotechnology #sc‐133098, RRID::AB_2193048) and CoxIV (1:20,000, Cell Signaling Technologies #4844, RRID:AB_2085427) overnight at 4°C. Membranes were then washed with PBST and probed with respective horseradish peroxidase‐conjugated secondary antibodies (GE Healthcare #NA931, RRID:AB_772210 and #NA934, RRID:AB_772206) at 1:2000 in blocking buffer for 1 hr at room temperature. Membranes were washed again with PBST and developed with Clarity Western ECL Kit (Bio‐Rad). Western blots were quantified using the Analyze Gels plugin on ImageJ.

### 2.10 Statistical analysis

Organ bacterial loads and IHC quantification were compared using the nonparametric Mann–Whitney *U* test. All other comparisons were made using an unpaired *t* test. *p* values <.05 were considered significant.

## 3 RESULTS

### 3.1 Androgens augment TGFβ1 production in the kidney

**FIGURE 1:**
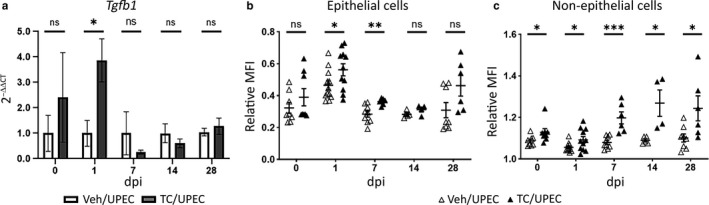
TGFβ1 levels are upregulated in the kidneys of TC‐treated mice. Female C57BL/6 mice were injected weekly with 150 mg/kg testosterone cypionate (TC) or vehicle starting at 5 weeks of age before transurethral inoculation of 10^7^ CFU of UPEC 2 weeks later. (a) Relative mRNA transcript levels of *Tgfb1* (normalized to *Gapdh*) in vehicle‐treated (open bars) or TC‐treated (gray bars) mice at various time points before and after UPEC inoculation; *n* = 4–8 mice per group, mean transcript level in vehicle‐treated mice set to 1 arbitrary unit at each time point. Relative mean fluorescence intensity (MFI) of TGFβ1 in (b) epithelial and (c) non‐epithelial cells, shown as a proportion of the MFI of live, CD45− cells, was determined by flow cytometry of whole kidney in vehicle‐treated (open triangles) or TC‐treated (filled triangles) mice; *n* = 4–12 mice per group. *p < .05, **p < .01, ***p < .001

**FIGURE 2:**
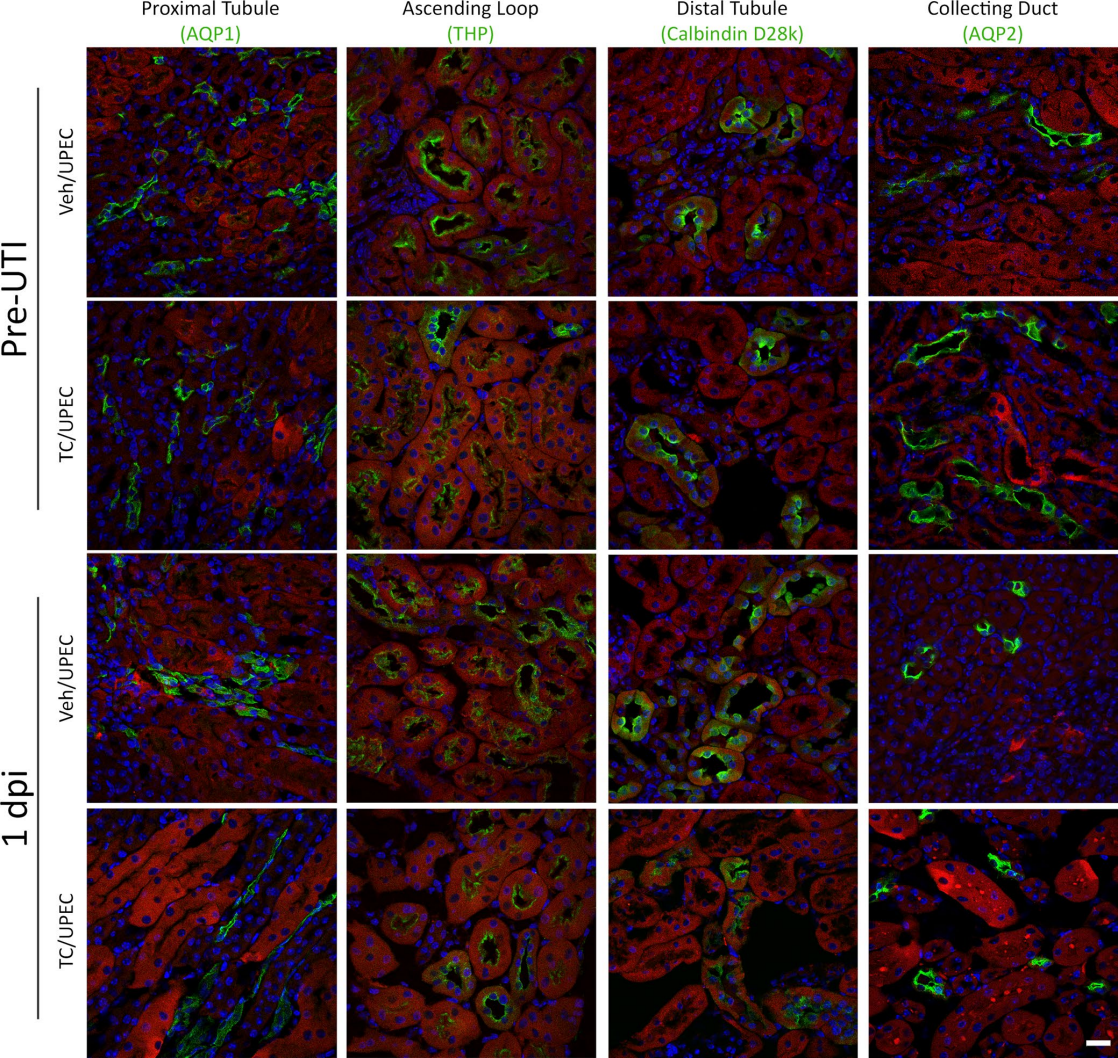
TGFβ1 is produced in selected tubular segments in both vehicle and TC‐treated mice. Immunofluorescence imaging of 8‐µm sections of fixed, frozen kidneys shows that the TGFβ1 (red in all images) is primarily produced in the distal tubule (green, calbindin D28k) and thick ascending loop (green, Tamm‐Horsfall protein [THP]), while TGFβ1 did not colocalize with the proximal tubule (green, aquaporin‐1 [AQP1]), or the collecting duct (green, aquaporin‐2 [AQP2]) in vehicle‐ or TC‐treated mice at the time of UPEC inoculation and 1 dpi. Scale bar represents 20 µm

To investigate the effect of androgens on renal fibrosis following pyelonephritis, female C57BL/6 mice were treated weekly with 150 mg/kg of testosterone cypionate (TC) or vehicle beginning 2 weeks before UPEC inoculation. Mice treated with TC had significantly higher levels of serum testosterone compared to vehicle‐treated mice, accompanied by mild but detectable increases in serum estradiol levels, presumably via aromatization (Figure S1). Consistent with our prior data showing more severe UTI outcomes in androgenized hosts, TC‐treated mice harbored significantly higher bladder and kidney bacterial loads at later time points (14 and 28 dpi), with a higher proportion of TC‐treated mice exhibiting high‐titer pyelonephritis (>10^4^ CFU; Figure S2). Elevation of circulating androgens increased both expression and production of TGFβ1 globally in the kidney. Transcript levels of *Tgfb1* in the whole kidney were slightly higher at the time of infection and were significantly higher in TC‐treated mice 1‐day post‐infection (Figure 1a). Flow cytometry analysis determined that at baseline (prior to initiation of UTI), increased TGFβ1 protein expression in TC‐treated mice was observed only in non‐hematopoietic (CD45−) cell populations comprising the renal parenchyma. Among these CD45− subsets, TGFβ1 production by renal epithelial cells (E‐cadherin+, CD45−) peaked early (1 dpi) in response to UPEC infection in both vehicle‐ and TC‐treated mice. TGFβ1 protein production by epithelial cells in the kidneys of TC‐treated mice was also increased compared to those of vehicle‐treated mice 1 and 7 dpi (Figure 1b). Immunofluorescence microscopy revealed TGFβ1 production throughout the nephron but primarily in the thick ascending loop and distal tubule in both vehicle and TC‐treated mice, both before and 1 day post infection, with no significant TGFβ1 production in the medulla (Figure 2, Figure S3). Meanwhile, TGFβ1 production by non‐epithelial cell subsets (CD45−, E‐cadherin−; comprising endothelial cells, vascular smooth muscle cells, podocytes, mesangial cells, fibroblasts, and activated myofibroblasts) was consistently higher in TC‐treated mice than in vehicle‐treated mice across all time points (0–28 dpi), steadily increasing over time only in TC‐treated mice (Figure 1c).

### 3.2 Increased early TGFβ1 perturbs levels of downstream fibrotic signaling factors

**FIGURE 3:**
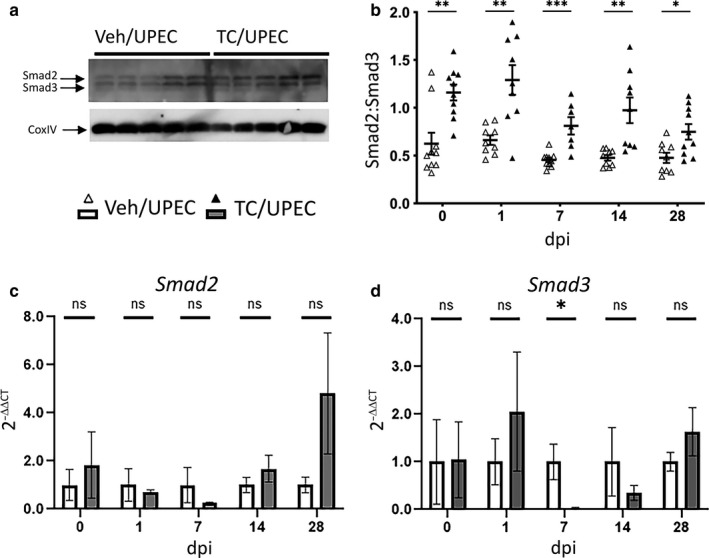
TC treatment alters the Smad2:Smad3 ratio throughout the course of infection. The ratio of Smad2 and Smad3 was determined by immunoblot (as exemplified with five individual vehicle‐ and TC‐treated mice 14 dpi) in panel (a) and quantified in (b), at the time of UPEC inoculation and at 1, 7, 14 and 28 dpi in whole‐kidney homogenates from vehicle‐treated mice (open triangles) or TC‐treated mice (filled triangles); *n* = 7–10 mice per group. The relative mRNA expression of (c) *Smad2* and (d) *Smad3* in whole kidney (normalized to *Gapdh*) was determined at the above time points in both vehicle‐treated (open bars) and TC‐treated (gray bars) mice; *n* = 4–8 mice per group. *p < .05, **p < .01, ***p < .001

In other global kidney injury models (e.g., unilateral ureteral obstruction [UUO]), increased TGFβ1 production in the kidney alters the ratio of transcription factors Smad2 and Smad3, favoring activation of fibrosis programs (Kim et al., 2005). In addition, testosterone treatment of cultured ovarian epithelial cells was shown to suppress Smad3 production (Kollara, Shathasivam, Park, Ringuette, & Brown, 2020). At the time points noted above after UPEC infection of C57BL/6 mice, we performed quantitative immunoblots on whole kidney homogenates for total Smad2 and Smad3 (Figure 3a). Kidneys of TC‐treated mice exhibited a higher Smad2:Smad3 ratio than vehicle‐treated mice, beginning before the onset of UTI and continuing throughout the course of infection (Figure 3b). Of note, we could not reliably detect phospho‐Smad2 and phospho‐Smad3 in these homogenates despite multiple approaches (see Discussion). By qPCR analysis of whole‐kidney RNA, *Smad2* expression in TC‐treated mice was statistically equivalent to that in vehicle‐treated mice across the course of infection (Figure 3c), while *Smad3* expression was significantly suppressed in TC‐treated mice at 7 dpi, recovering by 28 dpi (Figure 3d). These data indicate that androgen exposure facilitates Smad3 suppression in the infected kidney, thereby promoting fibrosis after infectious injury.

### 3.3 Androgen exposure augments TGFβ1‐driven myofibroblast activation

**FIGURE 4:**
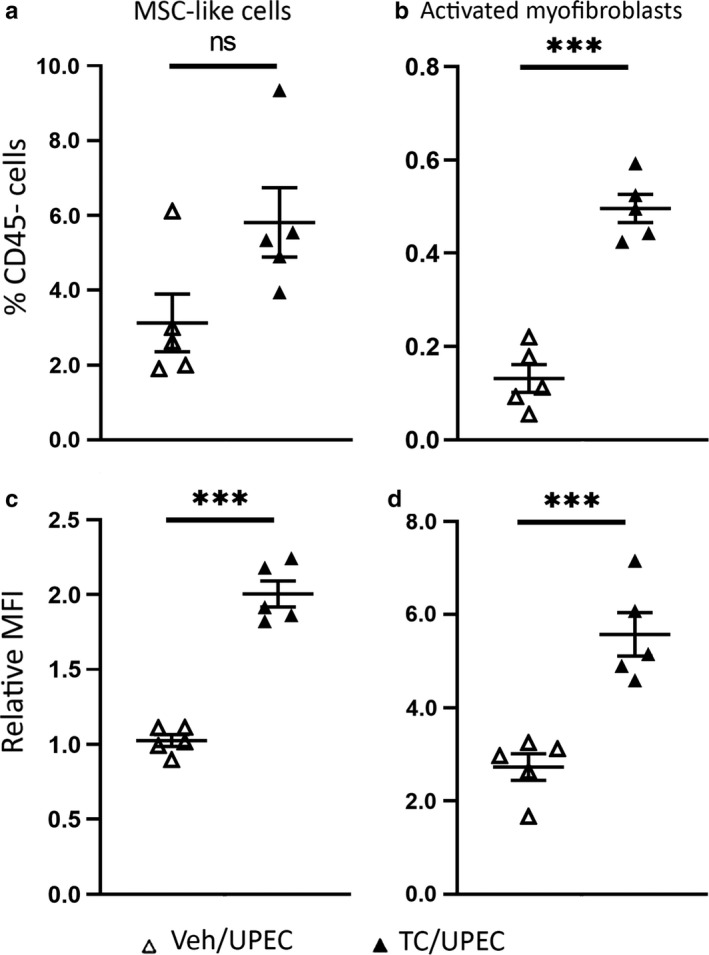
Increased TGFβ1 signaling primes TC‐treated mice for fibrosis by increasing myofibroblast activation. The populations of (a) MSC‐like cells and (b) activated myofibroblasts as a percentage of the total live, CD45− population at the time of infection were determined by flow cytometry in vehicle‐treated (open triangles) or TC‐treated mice (filled triangles). The MFI of TGFβ1 produced by (c) MSC‐like cells and (d) activated myofibroblasts as a proportion of the MFI of live, CD45− cells of vehicle‐ or TC‐treated mice was also determined by flow cytometry of whole kidney at the time of infection. *n* = 5 mice per group; ***p < .001

TGFβ1 signaling in other renal injury models recruits Gli1+ MSC‐like progenitor cells from the perivascular space and promotes the conversion of these cells and Gli1− kidney‐resident MSC‐like cells into activated myofibroblasts capable of depositing extracellular matrix components, including collagens (Asada et al., 2011; Kramann, Schneider, et al., 2015; Wan et al., 2012; Wu et al., 2013). We used flow cytometry to interrogate the effect of androgen exposure on the populations of kidney‐resident MSC‐like cells (CD45−, E‐cadherin−, PDGFRβ+, Gli1−; consisting of fibroblasts, mesangial cells, and pericytes (Boor, Ostendorf, & Floege, 2014; Klinkhammer, Floege, & Boor, 2018; Wu et al., 2013)) and activated myofibroblasts (CD45−, E‐cadherin−, PDGFRβ+, Gli1+, αSMA+, and Nestin+ (Humphreys, 2018; Kramann, Schneider, et al., 2015)) in the kidney before and throughout UPEC pyelonephritis. Prior to the onset of UTI, the kidneys of TC‐treated mice harbored slightly more MSC‐like cells (Figure 4a) and significantly more activated myofibroblasts (Figure 4b) than in vehicle‐treated mice. Moreover, the relative amount of TGFβ1 produced at baseline in both the MSC‐like cells (Figure 4c) and activated myofibroblasts (Figure 4d) was significantly higher in TC‐treated mice. This augmented recruitment of MSC‐like cells and activation of myofibroblasts, in conjunction with their increased TGFβ1 expression, suggests that androgen exposure “primes” these cell populations within the kidney for fibrotic signaling, even before renal injury occurs.

**FIGURE 5:**
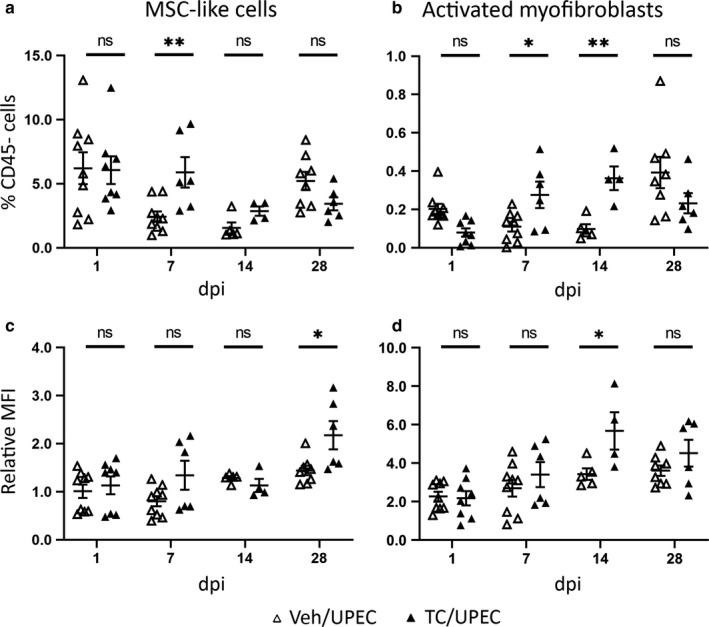
Androgen exposure influences fibroblast and myofibroblast activation and TGFβ1 production throughout the course of infection. The population of (a) MSC‐like cells and (b) activated myofibroblasts as a percentage of live, CD45− cells was determined by flow cytometry of whole kidney in vehicle‐treated (open triangles) or TC‐treated (filled triangles) mice at 1, 7, 14, and 28 dpi. The relative MFI of TGFβ1 production compared to live, CD45− cells in (c) MSC‐like cells and (d) activated myofibroblasts at the same time points was also determined by flow cytometry in vehicle‐ and TC‐treated mice. *n* = 4–12 mice per group; *p < .05, **p < .01

Over a time course following UPEC inoculation, MSC‐like cells were significantly more populous in androgenized mice only at 7 dpi (Figure 5a). Meanwhile, the population of activated myofibroblasts was significantly elevated in TC‐treated mice beginning 7 dpi (*p* = .0096 vs. TC‐treated mice at 1 dpi), while in vehicle‐treated mice, this population was elevated only much later (28 dpi; *p* = .0187 vs. vehicle‐treated mice at 14 dpi; Figure 5b). Of note, in uninfected mice treated with vehicle or TC and allowed to grow to ages equal to those 14 or 28 dpi (9‐ and 11‐week old, respectively), populations of MSC‐like cells resembled those in comparable UPEC‐infected mice (Figure S4a). In contrast, the activated myofibroblast population was not significantly different between uninfected vehicle‐ and TC‐treated mice (Figure S4b), indicating that the infectious injury was specifically required to induce myofibroblast activation.

Over time following UPEC infection, the amount of TGFβ1 produced by MSC‐like cells and activated myofibroblasts (on a per‐cell basis) increased in both TC‐ and vehicle‐treated mice (Figure 5c and d), similar to the time course of TGFβ1 transcription and production reported in other renal injury models (Kilari, Yang, Sharma, McCall, & Misra, 2018; Popova et al., 2010; Wu et al., 2013). In MSC‐like cells, TGFβ1 production was higher in TC‐treated mice than in vehicle‐treated mice only at 28 dpi (Figure 5c); this also occurred in uninfected TC‐treated mice of similar age (Figure S4c). TGFβ1 production in MSC‐like cells rose in TC‐treated mice over time (28 dpi vs. 14 dpi, *p* = .026; Figure 5c). Similarly, per‐cell TGFβ1 production by activated myofibroblasts in TC‐treated mice was significantly higher than in vehicle‐treated mice only at 14 dpi (Figure 5d), when the activated myofibroblast population was also comparatively greatest (Figure 5b). Immunofluorescence microscopy indicated that these activated myofibroblasts are the predominant cellular sources of TGFβ1 within the kidney 14 dpi (Figure S5). Although TC treatment of uninfected mice did significantly increase TGFβ1 production by activated myofibroblasts (Figure S4d), this effect of androgenization was amplified in UPEC‐infected mice (*p* = .026 vs. uninfected at 14 dpi; *p* = .039 at 28 dpi; Figure 5d). Taken together, these data indicate that the chief effect of androgen exposure is to enhance TGFβ1 production by multiple cell types within the kidney, leading to accelerated activation and proliferation of myofibroblasts upon subsequent UPEC‐induced injury.

### 3.4 Androgen‐enhanced myofibroblast activation correlates with scar formation

**FIGURE 6:**
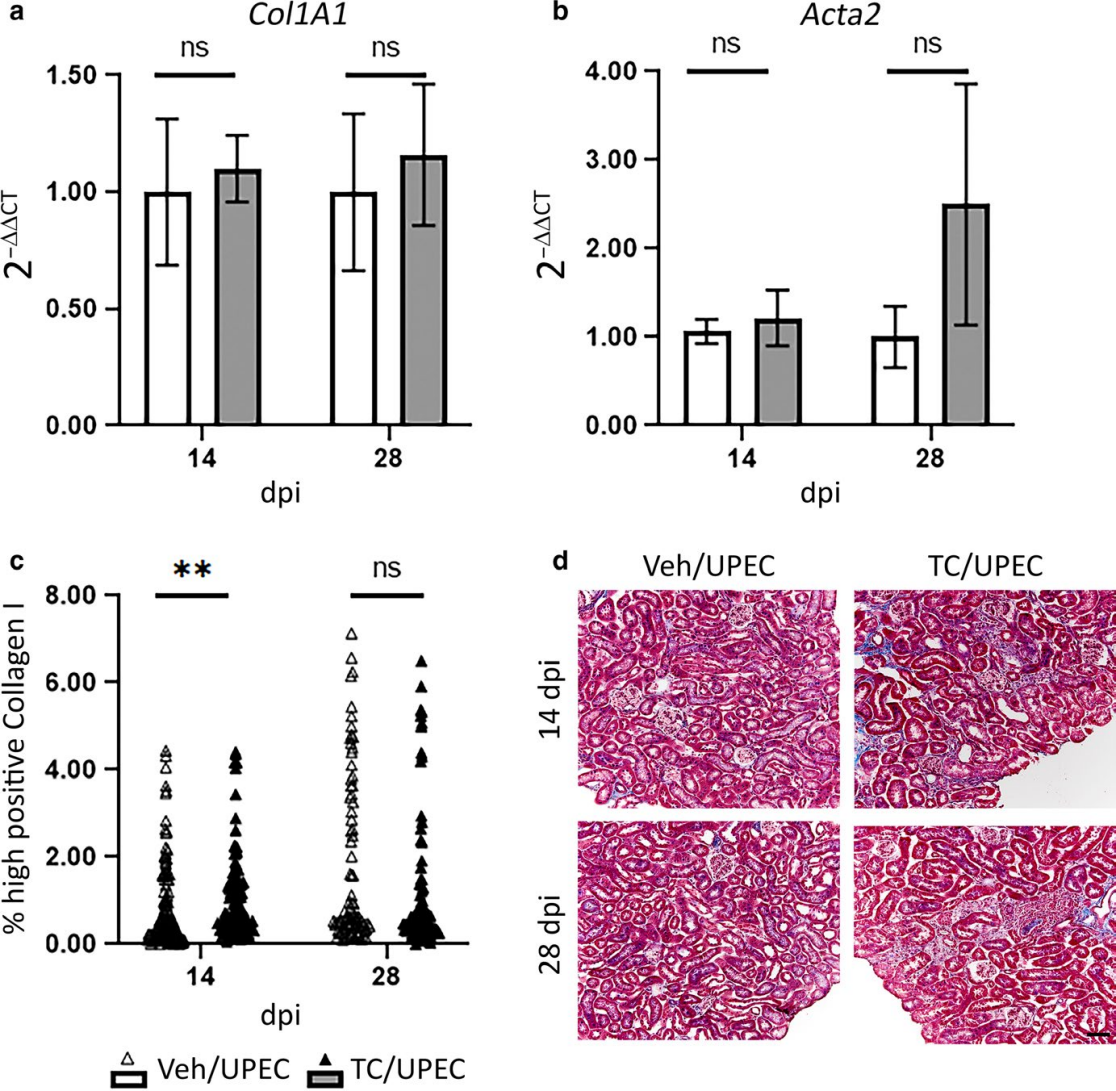
Increased myofibroblast activation in TC‐treated mice is associated with early and persistent inflammation and scarring in the renal cortex. Relative mRNA expression of (a) Collagen I (*Col1A1*) and (b) αSMA (*Acta2*) compared to GAPDH in vehicle‐treated (open bars) and TC‐treated (gray bars) at the time of UPEC inoculation and 1, 7, 14, and 28 dpi. *n* = 6–8 mice per group. (c) Collagen I deposition was quantified after immunohistochemical staining of 8‐µm sections of fixed, frozen kidneys of vehicle‐ and TC‐treated mice at 14 and 28 dpi. *n* = 102–136 random images from 12 to 15 sections from five mice per group. (d) 8‐µm sections of fixed, frozen kidneys of vehicle‐ or TC‐treated mice 14 and 28 dpi were stained with Gomori trichrome, highlighting collagen in blue. **p < .01; scale bar represents 50 µm

**FIGURE 7:**
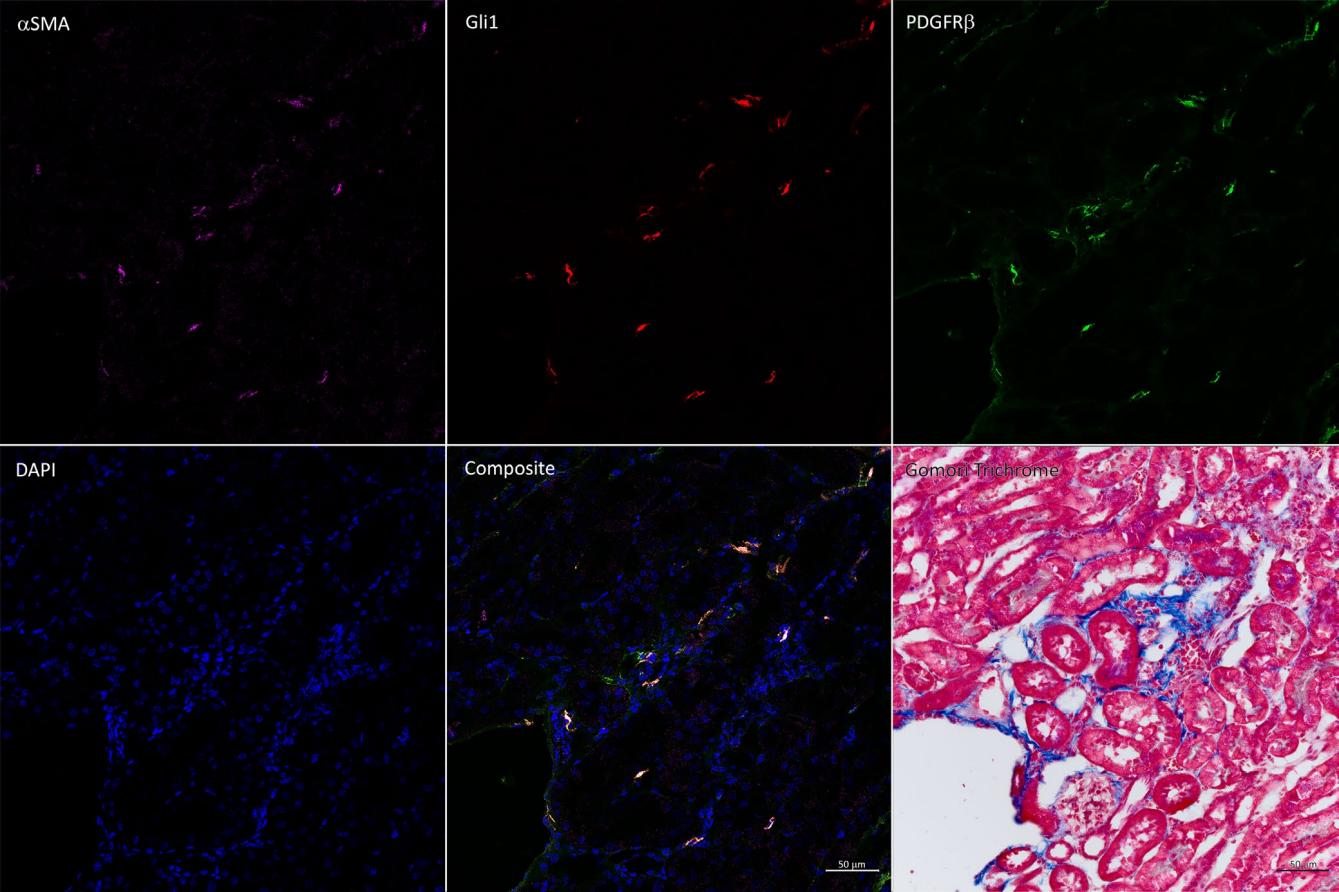
Activated myofibroblasts are localized to regions of scarring in TC‐treated mice. Immunofluorescence staining of 8‐µm sections of fixed, frozen kidneys from TC‐treated Gli1‐tdTomato mice 28 dpi show that activated myofibroblasts stain positively for αSMA+ (violet), Gli1+ (red), and PDGFRβ+ (green). Gomori trichrome staining of the adjacent section demonstrates that the activated myofibroblasts reside in areas of high collagen deposition and inflammation. Scale bar represents 50 µm

**FIGURE 8:**
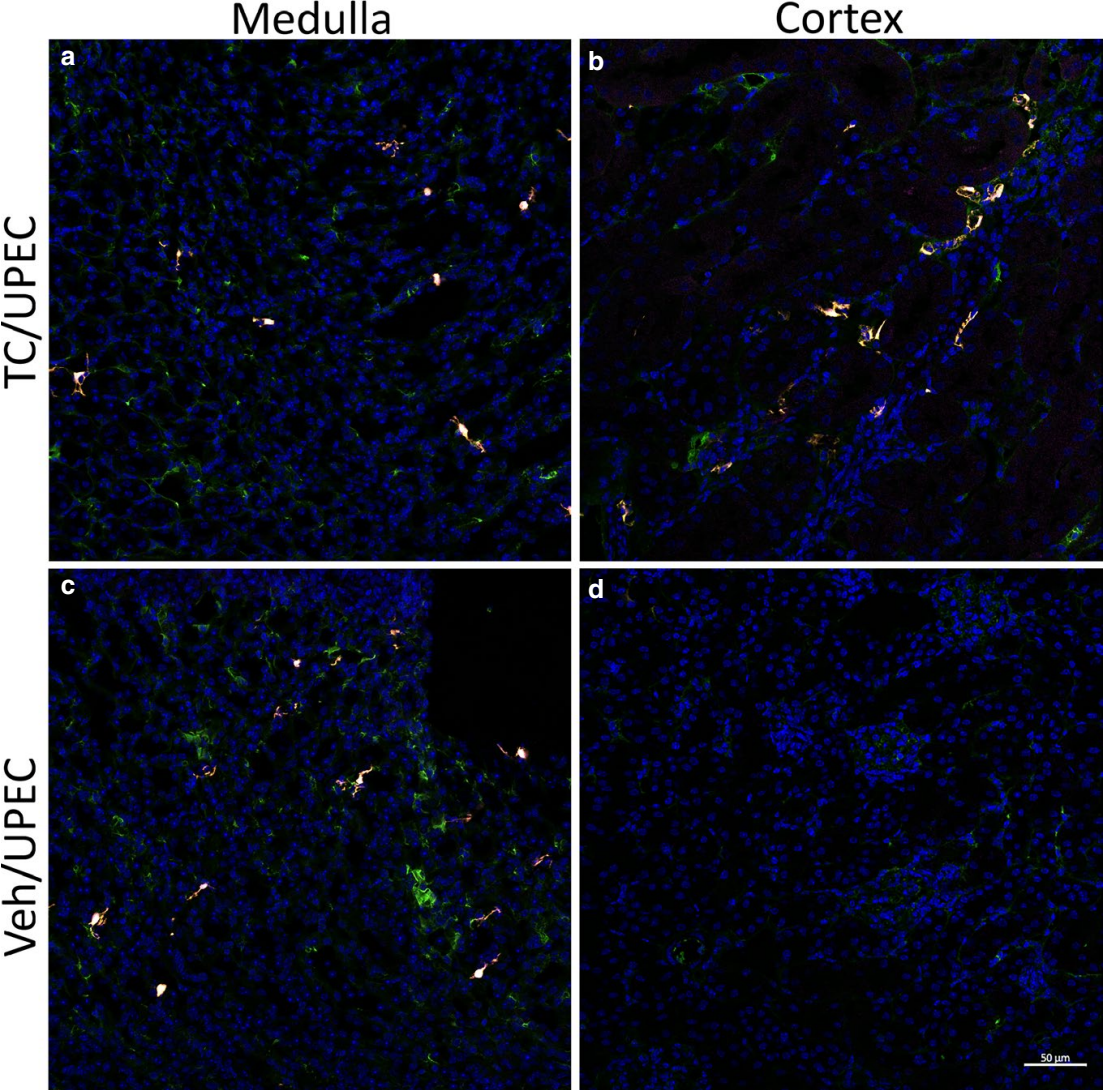
Activated myofibroblasts are localized to the renal cortex only in TC‐treated mice. Immunofluorescence imaging of 8‐µm sections of fixed, frozen kidneys from vehicle‐ or TC‐treated Gli1‐tdTomato mice 28 dpi shows that activated myofibroblasts (αSMA+ (violet), Gli1+ (red), and PDGFRβ+ (green)) and MSC‐like cells (PDGFRβ+ alone) are identified in the medulla in mice receiving either TC (a) or vehicle (c), whereas activated myofibroblasts are observed in the renal cortex only in TC‐treated mice (b), and not in vehicle‐treated mice (d). Scale bar represents 50 µm

Prior work indicates that local myofibroblast activation drives fibrotic scarring in non‐infectious, global renal injury models (Humphreys et al., 2010; Kramann, Schneider, et al., 2015; Picard, Baum, Vogetseder, Kaissling, & Le Hir, 2008; Sato & Yanagita, 2017). In comparison, our pyelonephritis model features regional injury and scarring that is comparatively limited in scope; as a result, analysis of our whole‐kidney homogenates did not reveal increased *Col1A1* (collagen I) and *Acta2* (α‐smooth muscle actin) transcription in TC‐treated mice (Figure 6a and b). Quantified IHC staining indicated an increase in collagen I deposition in the kidneys of TC‐treated mice at 14 dpi (Figure 6c), while collagen I staining had equalized between groups by 28 dpi. Furthermore, TC‐treated mice developed regions of focal scarring visible (by Gomori trichrome staining) in the renal cortex by 14 dpi and persisting through 28 dpi; such regions were not identified in vehicle‐treated mice (Figure 6d). We performed analogous infections in TC‐treated Gli1‐tdTomato^+^ mice, and the resulting scars contained streaks of activated myofibroblasts, which stained positively for αSMA, Gli1 (via the Tomato fluorophore), and PDGFRβ, and were surrounded by areas of dense collagen deposition (Figure 7). Activated myofibroblasts are normally present (in the absence of injury) in the renal medulla and near the vasculature (Kramann, Schneider, et al., 2015); in our infection model, these cells were visible in the medulla in both TC‐ and vehicle‐treated mice (Figure 8a and c). However, they were found in cortical locations only in TC‐treated mice (Figure 8b and d), consistent with the localization of renal scars in these mice.

**FIGURE 9:**
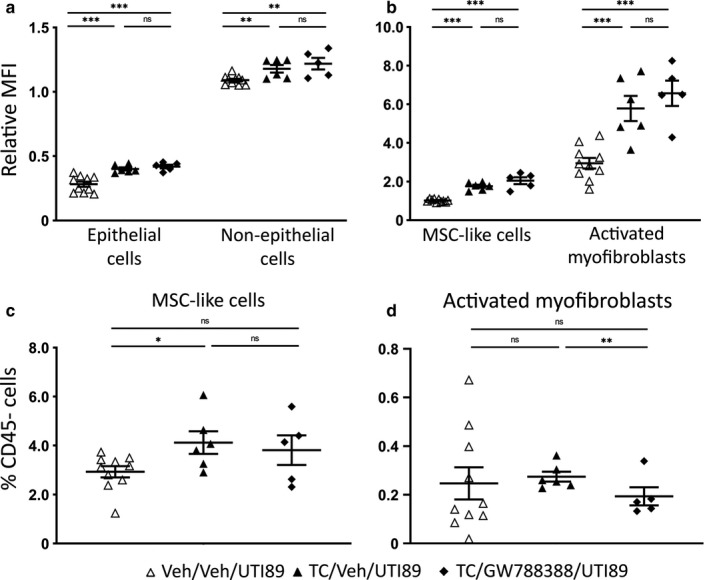
TGFβR1 inhibition limits androgen‐enhanced myofibroblast activation. TGFβ1 production (MFI compared to live, CD45− cells) was measured 7 dpi in mice receiving vehicle/vehicle (open triangles), TC/vehicle (filled triangles), or TC/GW788388 (filled diamonds) in (a) epithelial and non‐epithelial cells or (b) MSC‐like cells and activated myofibroblasts. The relative population of (c) MSC‐like cells and (d) activated myofibroblasts as a percentage of live, CD45− cells was also determined in these groups. *n* = 5–10 mice per group; *p < .05, **p < .01, ***p < .001

To further test whether androgen‐enhanced TGFβ1 was driving myofibroblast activation and scarring following UPEC pyelonephritis, we administered a specific TGFβ1 receptor antagonist (GW788388; (Gellibert et al., 2006; Lagares et al., 2010; McMillin et al., 2019; Petersen et al., 2008)) to TC‐treated mice for 7 days, beginning the day prior to UPEC inoculation. As seen in our initial experiments (Figure 1), androgen treatment (vs. vehicle) increased TGFβ1 production by CD45− cell populations 7 dpi; as expected, this increase was unaltered by GW788388 treatment (Figure 9a and b). Similarly, MSC‐like cell numbers were increased by androgen treatment (vs. vehicle) and not further changed by GW788388 treatment (Figure 9c). However, GW788388 treatment did significantly diminish the activated myofibroblast population in androgenized kidneys (Figure 9d), consistent with a model in which TGFβ1 mediates androgen‐enhanced renal scarring following UPEC infection.

## 4 DISCUSSION

In this work, we demonstrate that renal fibrosis after experimental UPEC pyelonephritis is driven by TGFβ1 and enhanced by androgens. Specifically, androgen exposure augments the production of fibrotic signaling factors (including TGFβ1) globally in the kidney, and alters the relative expression of the downstream transcription factors Smad2 and Smad3. In other models of renal fibrosis following non‐infectious insult, MSC‐like cell populations residing in the kidney and recruited from circulating precursors become activated myofibroblasts after local exposure to TGFβ1, subsequently depositing extracellular matrix components around the injured area that ultimately form a scar (Humphreys, 2018; Meng, Nikolic‐Paterson, & Lan, 2016). Here, we show that an analogous process underlies scarring after UPEC pyelonephritis, and we delineate the cellular and molecular mechanisms that are accelerated in the presence of androgens.

We previously showed that androgen exposure was linked to high‐titer pyelonephritis and significant renal scarring in C3H/HeN mice (Olson et al., 2016), which feature vesicoureteral reflux as a risk factor for upper‐tract UTI. In the present work, we utilized non‐refluxing C57BL/6 mice, which develop pyelonephritis of more modest severity after UPEC inoculation of the bladder. As we reported in the C3H background (Olson et al., 2016, 2018), androgen exposure was here found also to increase the rate of high‐titer pyelonephritis in C57BL/6 mice. Compared with the extensive scars formed in UPEC‐infected C3H mice (Olson et al., 2017), the BL/6 background enabled us to model of more limited renal scar formation, without overwhelming inflammation, and offers many more readily available genetic tools to enable future investigation.

Our qPCR and flow cytometric analyses showed that global TGFβ1 levels in the kidney were increased in androgenized mice even in the absence of injury. Androgenized mice exhibited corresponding increases in the population of MSC‐like cells and activated myofibroblasts residing in the kidney before infectious insult. These data indicate that androgen exposure itself promotes an environment that is primed for an enhanced fibrotic response should an injury occur. Indeed, male sex is reported as a risk factor for poor renal outcome (Eriksen & Ingebretsen, 2006; Evans et al., 2005; Iseki, Iseki, Ikemiya, & Fukiyama, 1996). Epidemiologic data from the United States, Europe and Japan show that kidney disease progresses faster in men than in women, resulting in men having a 60% greater incidence of end‐stage kidney disease (Centers for Disease Control & Prevention, 2019; Gretz, Zeier, Geberth, Strauch, & Ritz, 1989; Neugarten & Golestaneh, 2013).

Once UPEC infection began, the TGFβ1 signaling cascade was persistently more active in the androgenized host. *Tgfb1* transcription was increased in TC‐treated mice early in infection, with elevated TGFβ1 production observed in TC‐treated mice was most evident in epithelial cells, predominantly in the distal nephron, earlier in infection (up to 7 dpi) and then in non‐epithelial (CD45−, E‐cadherin−) cell types later in infection (7–28 dpi). This sequence is logical, as renal epithelial surfaces are likely contacted earliest by UPEC during ascending infection (Li et al., 2017; Olson et al., 2018). Thus, epithelial TGFβ1 signaling is likely the initiator of the fibrotic response to infection; in other models, TGFβ1 signaling from epithelial cells alone is sufficient to drive a fibrotic response to injury (Gentle et al., 2013; Olson et al., 2018).

The Smad2:Smad3 ratio was also consistently higher in the kidneys of TC‐treated mice than in vehicle‐treated mice throughout the course of infection, apparently driven primarily by suppression of *Smad3*. Although phosphorylation of Smad2 and Smad3 is required for these transcription factors to translocate to the nucleus and promote transcription of ECM components (Meng et al., 2016), we were unable to successfully quantify phospho‐Smad2/3 in the whole kidney despite several approaches. In contrast to other experimental renal injuries that are global (e.g., UUO), experimental pyelonephritis is a localized process, affecting only a limited portion of the kidney (Olson et al., 2016, 2017, 2018); as outlined above, this is particularly true in the BL/6 host and thereby limits our ability to discern changes in such analytes through analysis of whole‐kidney homogenates. Further work will need to be done to demonstrate phospho‐Smad2/3 alterations at the cellular level in scarred areas, rather than globally throughout the kidney. However, an altered total Smad2:Smad3 ratio was also shown in vitro to be a hallmark of fibrotic signaling (Kim et al., 2005), lending support to our conclusion that these transcription factors are key mediators of the post‐pyelonephritic scarring program.

Ultimately, TGFβ1 signaling in the infected kidney promotes the recruitment of precursors and the activation of myofibroblasts following the initiation of UTI. These αSMA+, Gli1+, PDGFRβ + cells are visible in the post‐pyelonephritic scar as it forms and matures, and they become the predominant producers of TGFβ1 during resolving infection. Activated myofibroblasts were observed in the medulla independent of androgen exposure, while myofibroblasts within collagen‐rich cortical scars were observed only in the androgenized host. Global collagen I deposition (by IHC) was enhanced in TC‐treated mice at 14 dpi, but matched in vehicle‐treated mice by 28 dpi, indicating that the acceleration of myofibroblast activation in the androgenized host mediated a pro‐scarring response earlier in the course of infection. Moreover, administration of a TGFβ receptor antagonist did not alter global TGFβ1 production in the kidney early in infection, but did result in reduction of the activated myofibroblast population in the injured, androgenized (and therefore scar‐susceptible) host. Along these lines, a detailed understanding of the molecular and cellular basis of post‐infectious scar formation, as well as hormonal and other influences on this injury response, may illuminate specific interventions to mitigate scarring after infection, promote functional parenchymal healing, and reduce long‐term complications.

## CONFLICT OF INTEREST

D.A.H. serves on the Board of Directors for BioVersys AG, Basel, Switzerland. All other authors have no potential conflicts to declare.

## Supporting information



Supplementary Material
