# Supplementary material for: TGFβ1 orchestrates renal fibrosis following Escherichia coli pyelonephritis
Source: Physiol Rep. 2020 Mar 30;8(6):e14401. doi: 10.14814/phy2.14401 (PMC7104652; doi:10.14814/phy2.14401)
Supplement: Supplementary file 1 — Supplementary Material [file PHY2-8-e14401-s001.pdf]

AJP-Renal Physiology

Supplemental Information

TGFβ1 orchestrates renal fibrosis following *Escherichia coli* pyelonephritis

Teri N. Hreha, Christina A. Collins, Allyssa L. Daugherty, Joy Twentyman, Nitin Paluri, and David A. Hunstad

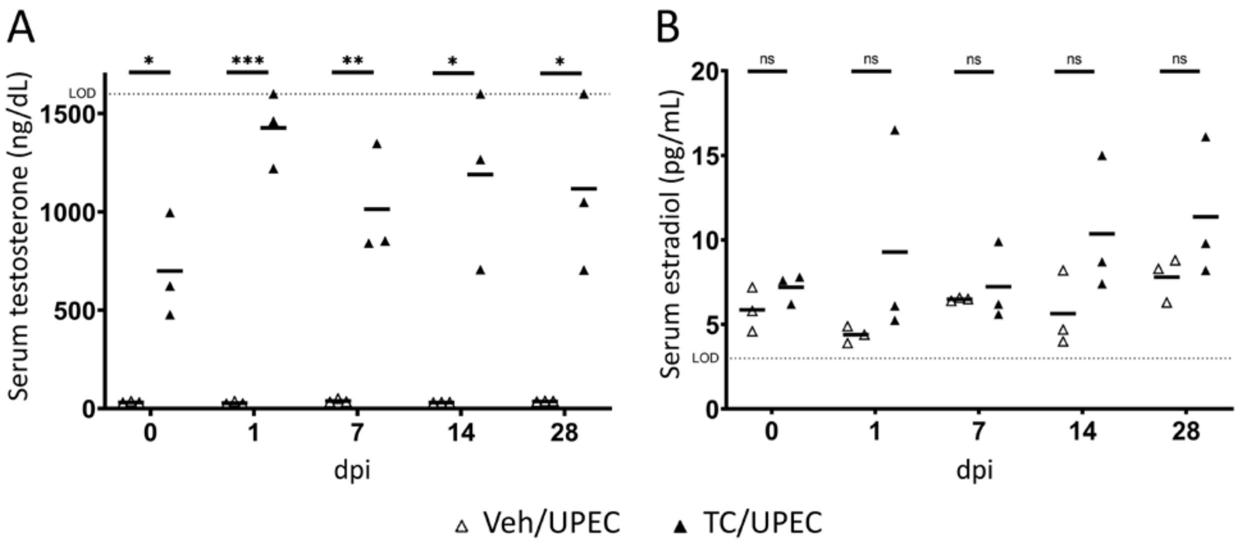

Figure S1: TC treatment increases serum testosterone levels with no significant effect on estradiol. Female C57BL/6 mice were injected weekly with 150 mg/kg testosterone cypionate (TC; filled triangles) or vehicle (Veh; open triangles) starting at 5 weeks of age, before transurethral inoculation with 10^7 CFU of UPEC 2 weeks later. Serum was collected through cardiac puncture at the time of sacrifice and analyzed for testosterone (A) or estradiol (B) levels via enzyme immunoassay at 0, 1, 7, 14 and 28 days post infection. Dotted lines indicate the limit of detection (LOD) for each assay. n = 3 per group; **P* < 0.05, ***P* < 0.01, ****P* < 0.001.

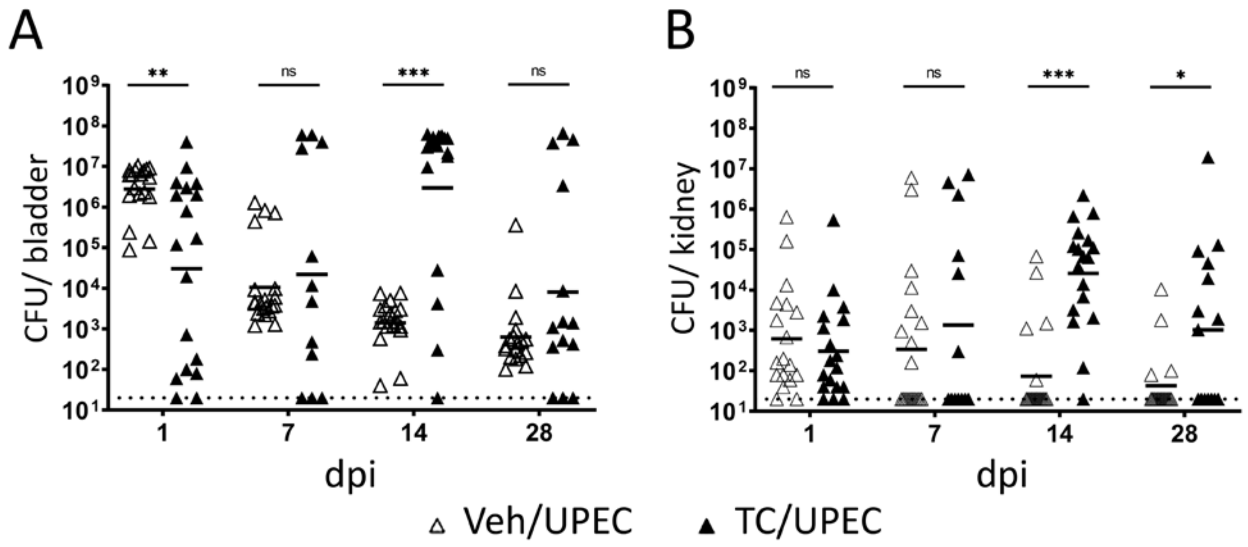

Figure S2: TC treatment increases the prevalence of chronic cystitis and pyelonephritis. Bacterial loads (CFU) per bladder (A) and kidney (B) were assessed by serially diluting organ homogenates from vehicle-treated (open triangles) or TC-treated (filled triangles) mice at 1, 7, 14 and 28 days post UPEC inoculation. Dotted line indicates limit of detection. n = 11-20 mice per group; *$P < 0.05$, **$P < 0.01$, ***$P < 0.001$.

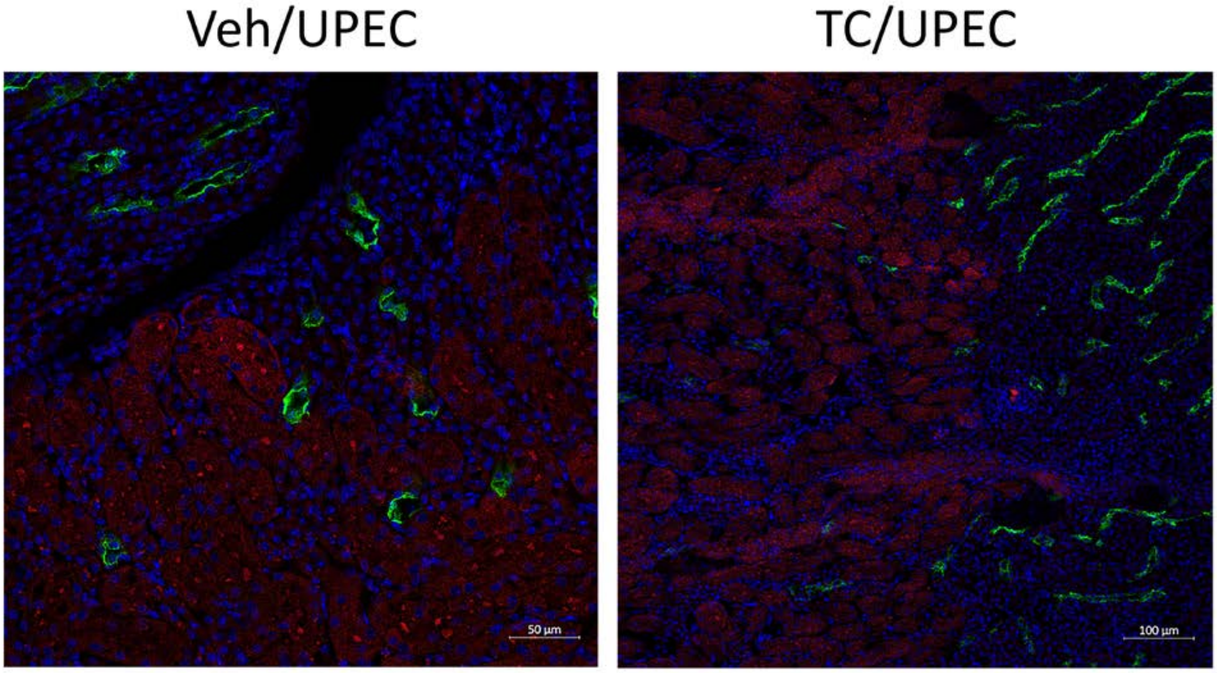

Figure S3: TGFB1 staining is present in the cortex, but not the medulla in vehicle-treated and TC-treated mice. Immunofluorescent staining of TGFβ1 (red), collecting duct (AQP2, green) and nuclei (blue) in 8-µm sections of fixed, frozen kidenys from vehicle (Veh) or testosterone cypionate (TC)-treated mice stain postively for TGFβ1 in the cortex, but not in the medulla at 1 dpi. Scale bar represents 50 µm for Veh, 100 µm for TC.

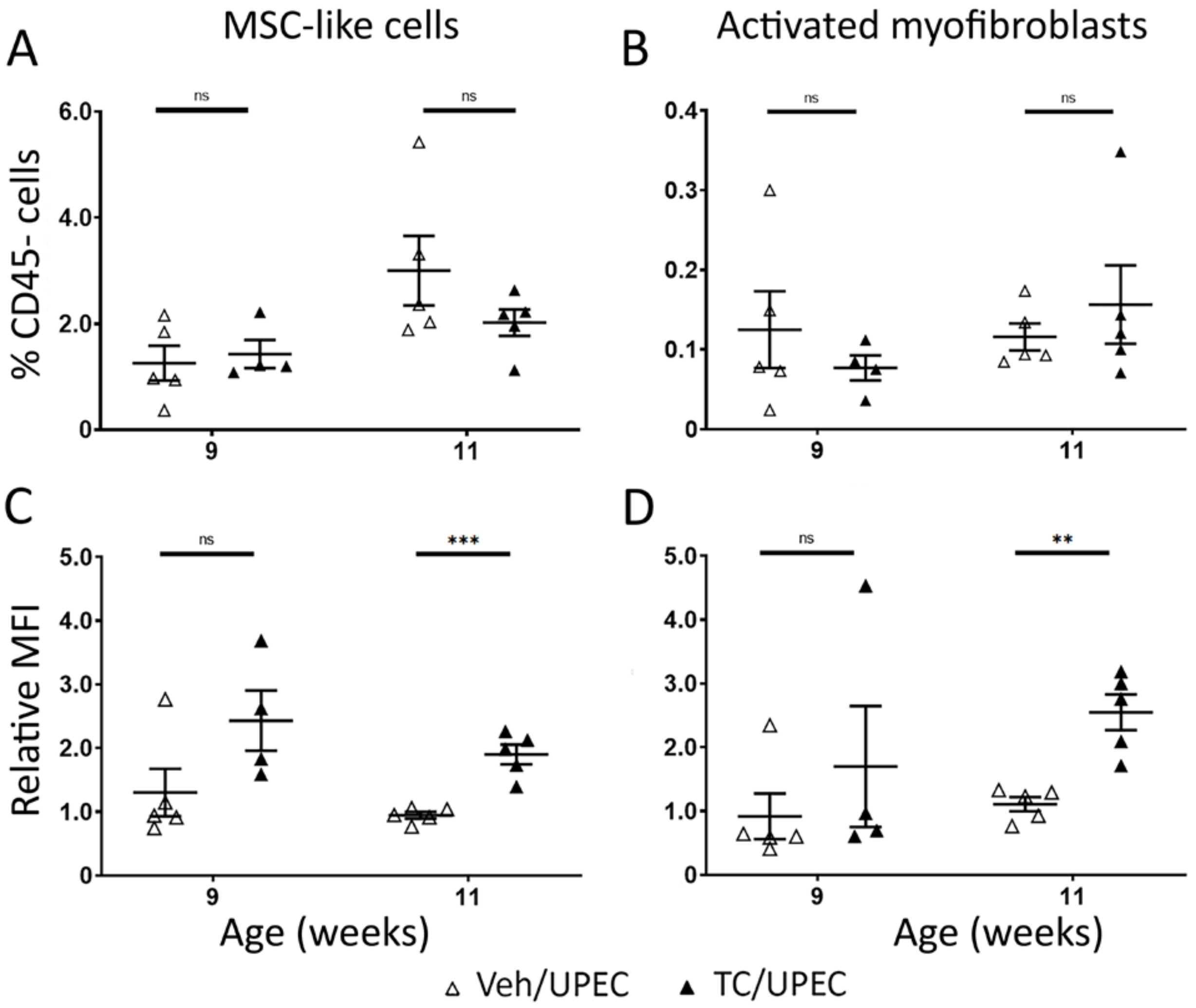

Figure S4: Androgen exposure alone is insufficient to induce myofibroblast activation. The population of (A) MSC-like cells and (B) activated myofibroblasts as a percentage of live, CD45- cells was determined by flow cytometry in uninfected vehicle-treated (open triangles) and TC-treated (closed triangles) mice at 9 and 11 weeks of age. The relative mean fluorescence intensity (MFI) of TGFβ1 compared to the MFI in live, CD45- cells of (C) MSC-like cells and (D) activated myofibroblasts in vehicle and TC-treated mice was determined by flow cytometric analysis of whole kidney. n = 4-5 mice per group; $^{**}P < 0.01$, $^{***}P < 0.001$.

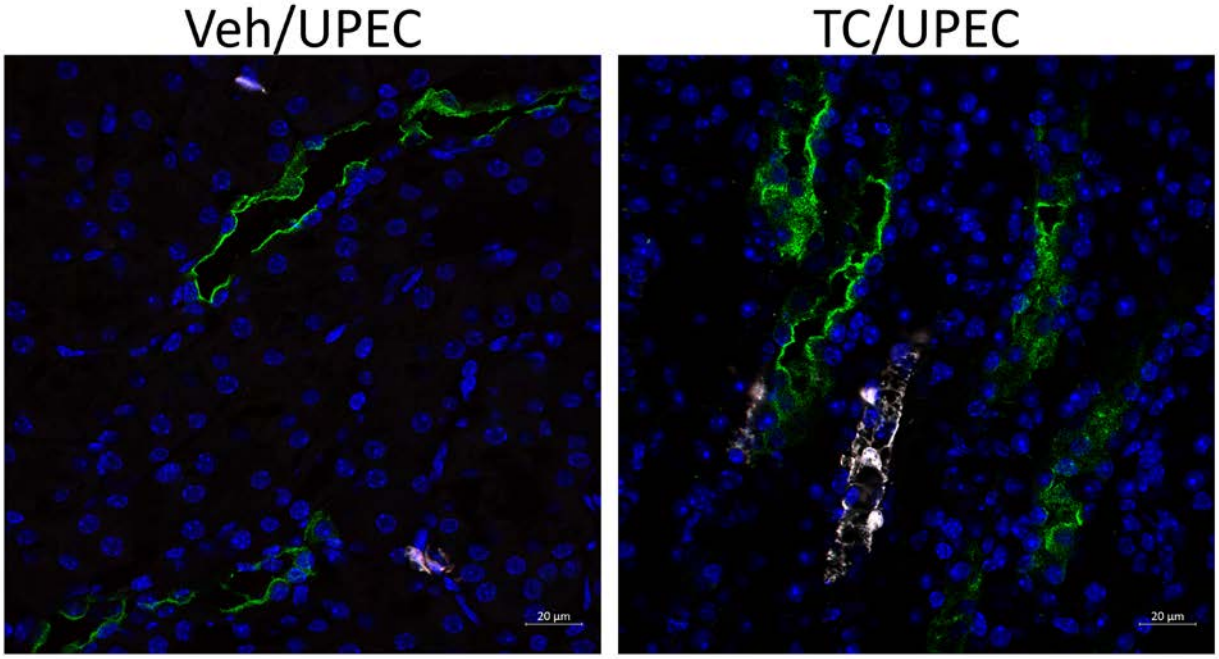

Figure S5: Activated myofibroblasts are the primary producers of TGFβ1 within the infected kidney 14 dpi. 8-µm sections of fixed, frozen kidneys from vehicle (Veh) or testosterone cypionate (TC)-treated Gli1-TdTomato mice indicate that Gli1+ activated myofibroblasts (cyan) stain positively for TGFβ1 (red), whereas TGFβ1 staining was not observed in the collecting duct (AQP2+, green) at 14 dpi. Scale bar represents 20 µm.

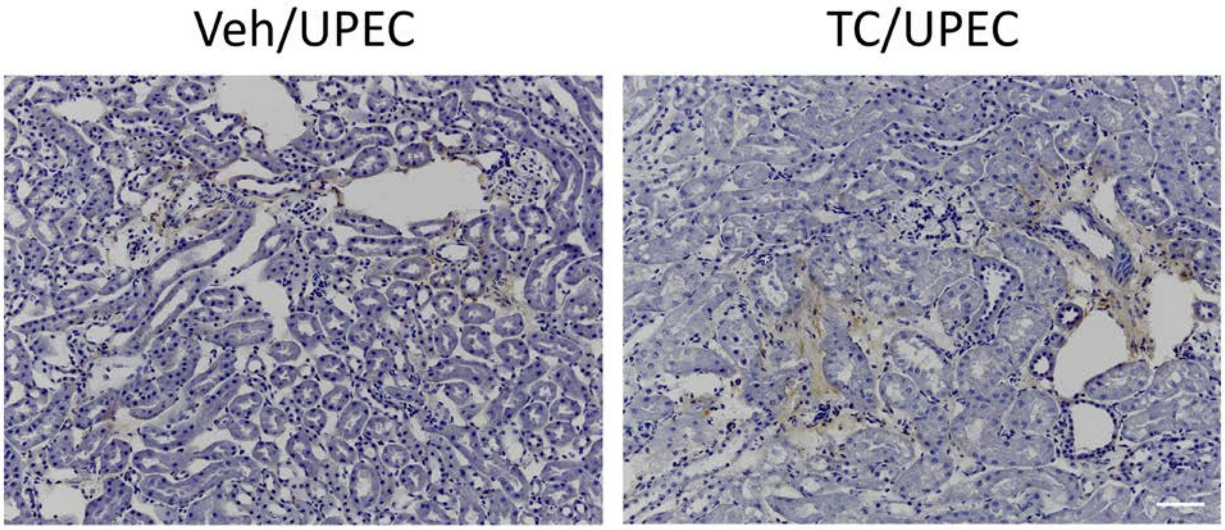

Figure S6: Representative collagen I IHC images used for quantitative analysis. 8-µm sections of fixed, frozen kidney from vehicle (Veh) and testosterone cypionate (TC)-treated mice were stained for collagen I and counterstained with hematoxylin. Scale bar represents 50 µm.

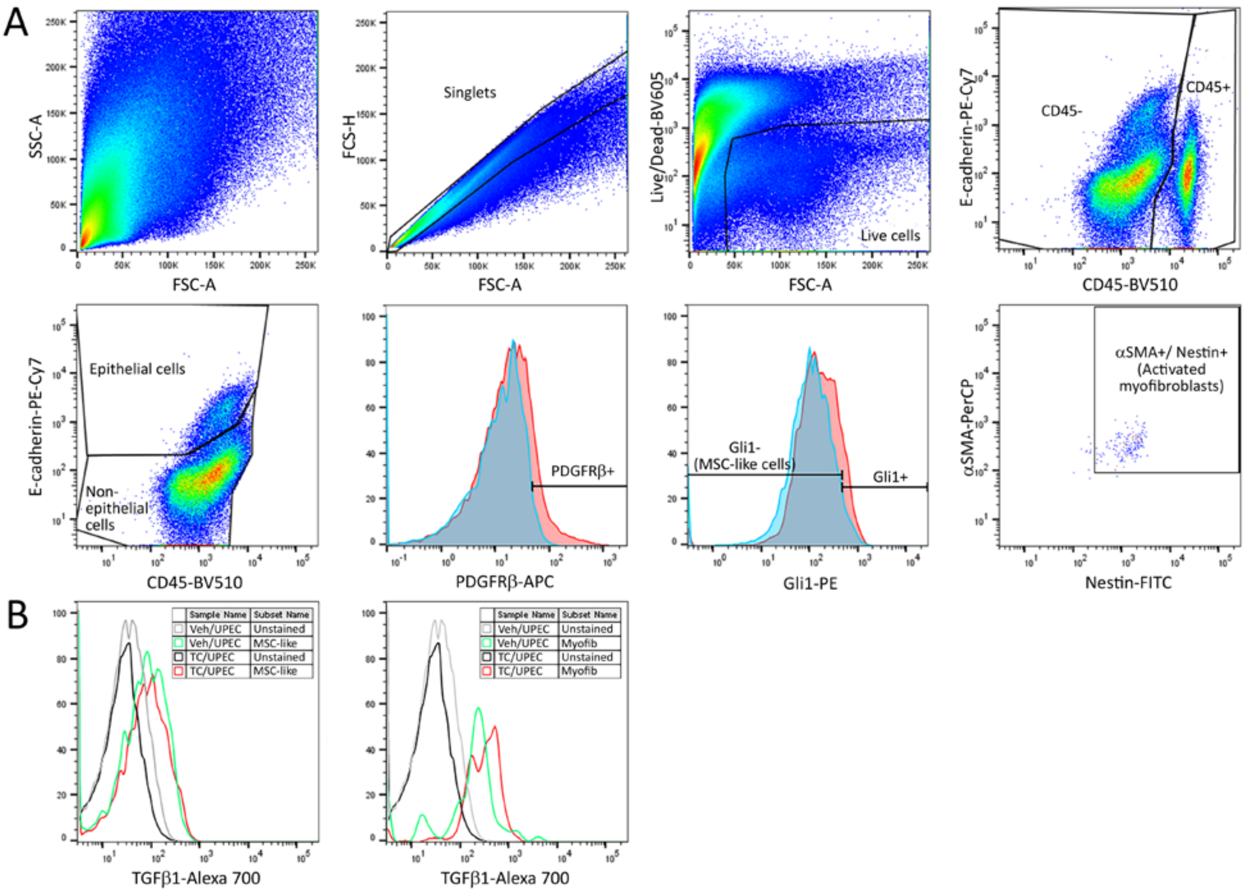

Figure S7: Representative flow cytometry analysis. (A) Representative gating scheme used for flow cytometry analysis to isolate epithelial cells (CD45−, E-cadherin+), non-epithelial cells (CD45−, E-cadherin−), MSC-like cells (CD45−, E-cadherin−, PDGFRβ+) and activated myofibroblasts (CD45−, E-cadherin−, PDGFRβ+, Nestin+, αSMA+) from the kidney. (B) Representative mean fluorescence intensities of TGFβ1 production in MSC-like cells and activated myofibroblasts in vehicle- or TC-treated mice compared to unstained kidney lysate of each treatment group.

Table S1. Primers used in this study

Gene	Protein	Forward Primer Sequence	Reverse Primer Sequence
Gapdh	GAPDH	TGTTACCAACTGGGACGACA	GGGGTGTTGAAGGTCTCAAA
Tgfb1	TGFβ1	TGATACGCCTGAGTGGCTGTCT	CACAAGAGCAGTGAGCGCTGAA
Col1A1	Collagen I	CCTCAGGGTATTGCTGGACAAC	CAGAAGGACCTTGTTTGCCAGG
Acta2	αSMA	CCCCTGAAGAGCATCGGACA	TGGCGGGGACATTGAAGGT
Smad2	Smad2	ACTAACTTCCCAGCAGGAAT	GTTGGTCACTTGTTTCTCCA
Smad3	Smad3	CCACGCAGAACGTCAACA	TTGAAGGCGAACTCACACAG
Gli1	Gli1	CCTGGTGGCTTTCATCAACT	ACACAGGGCTGGACTCCATA
